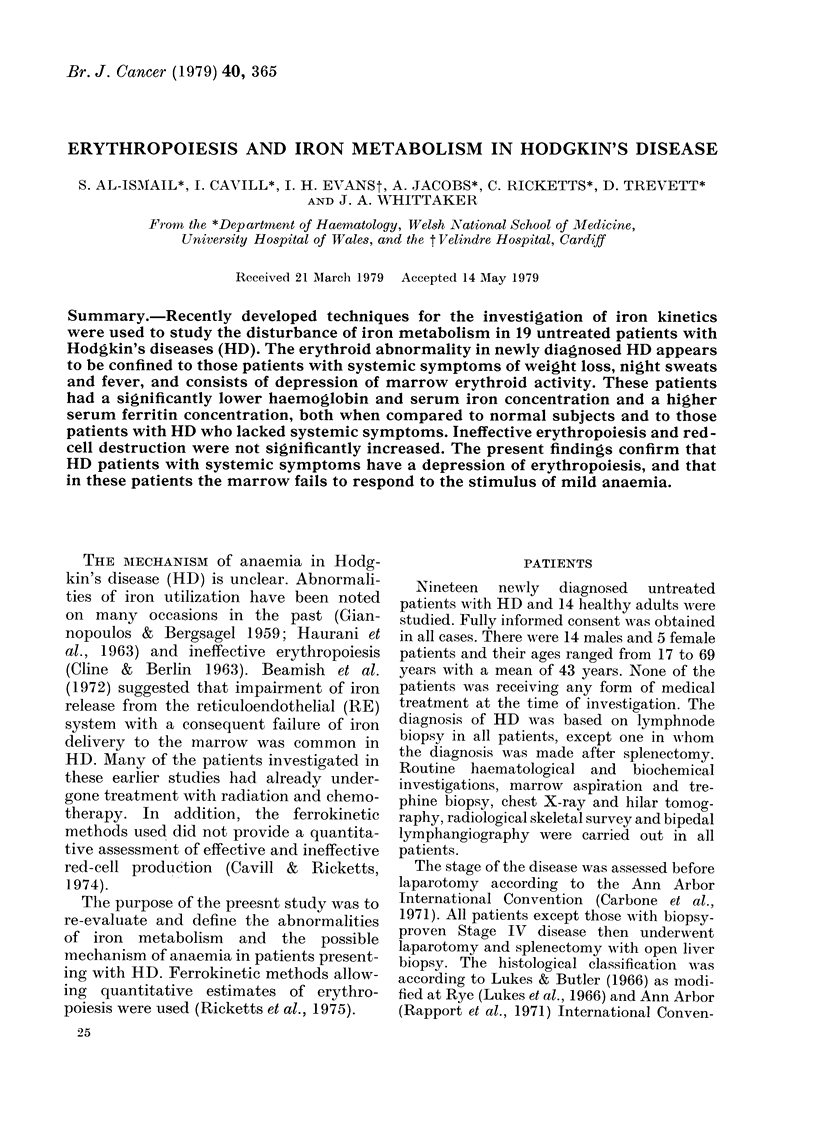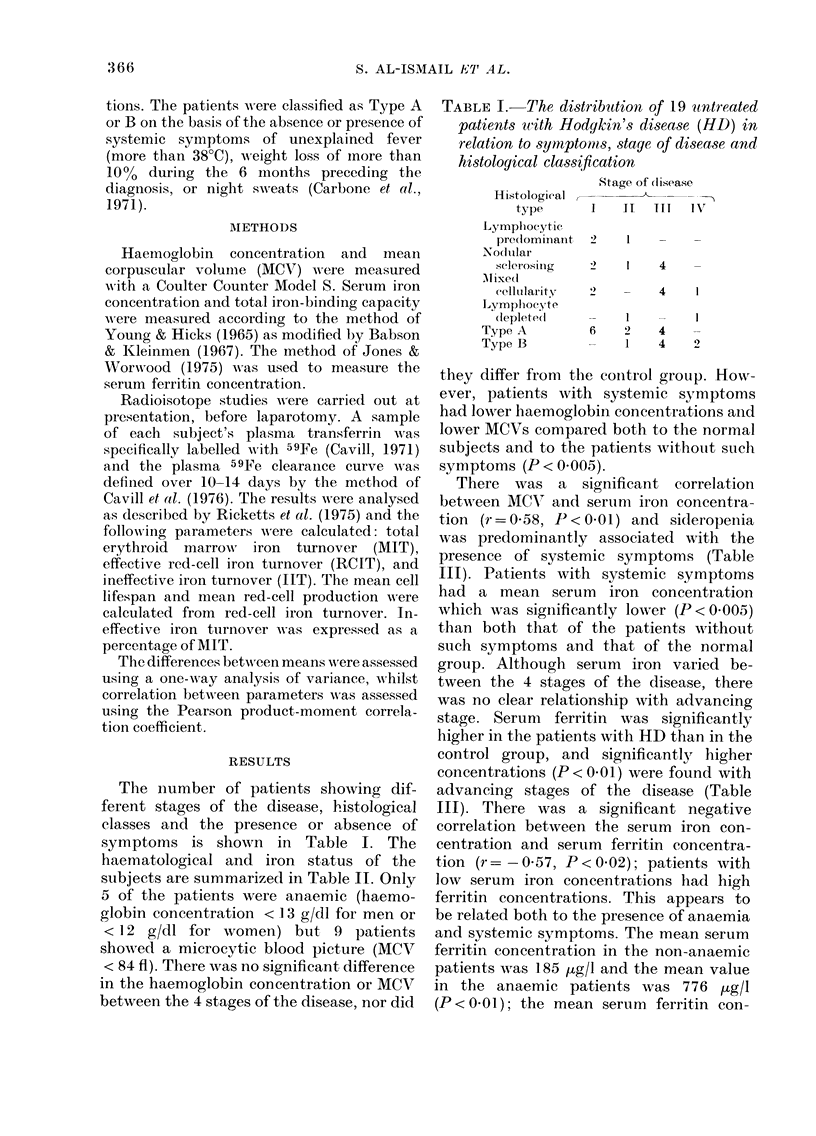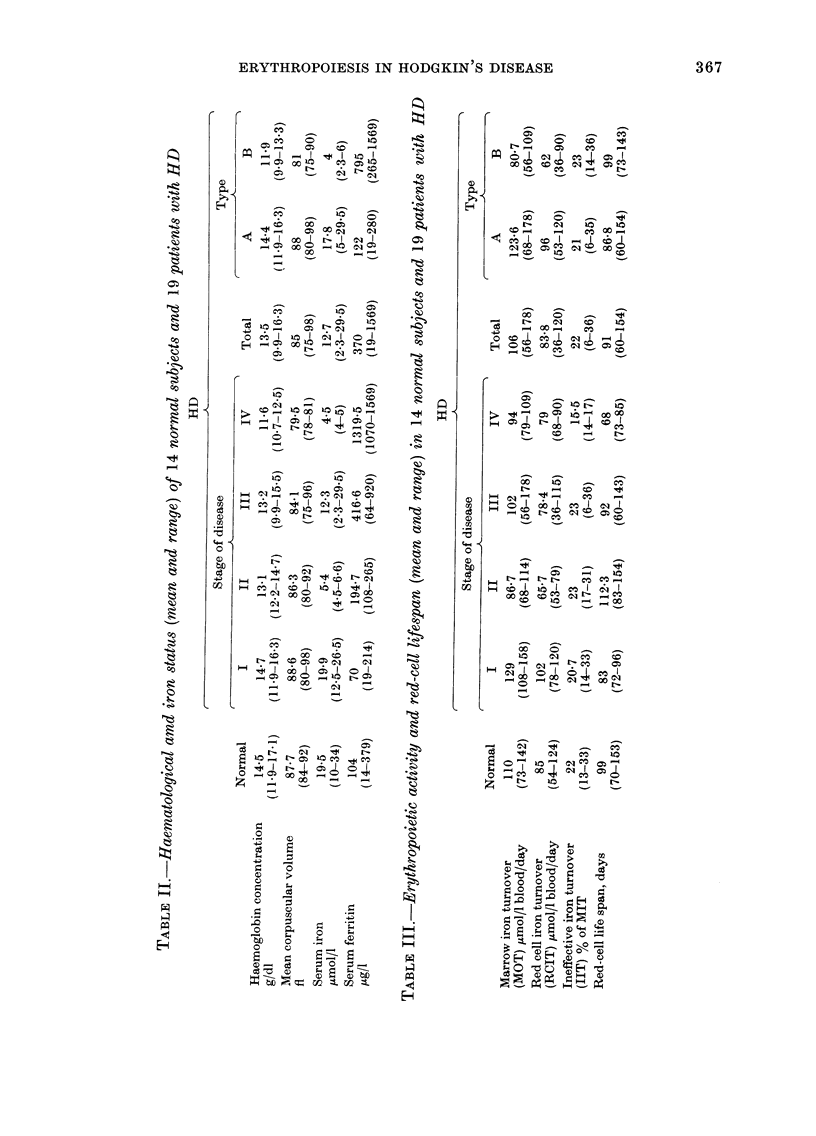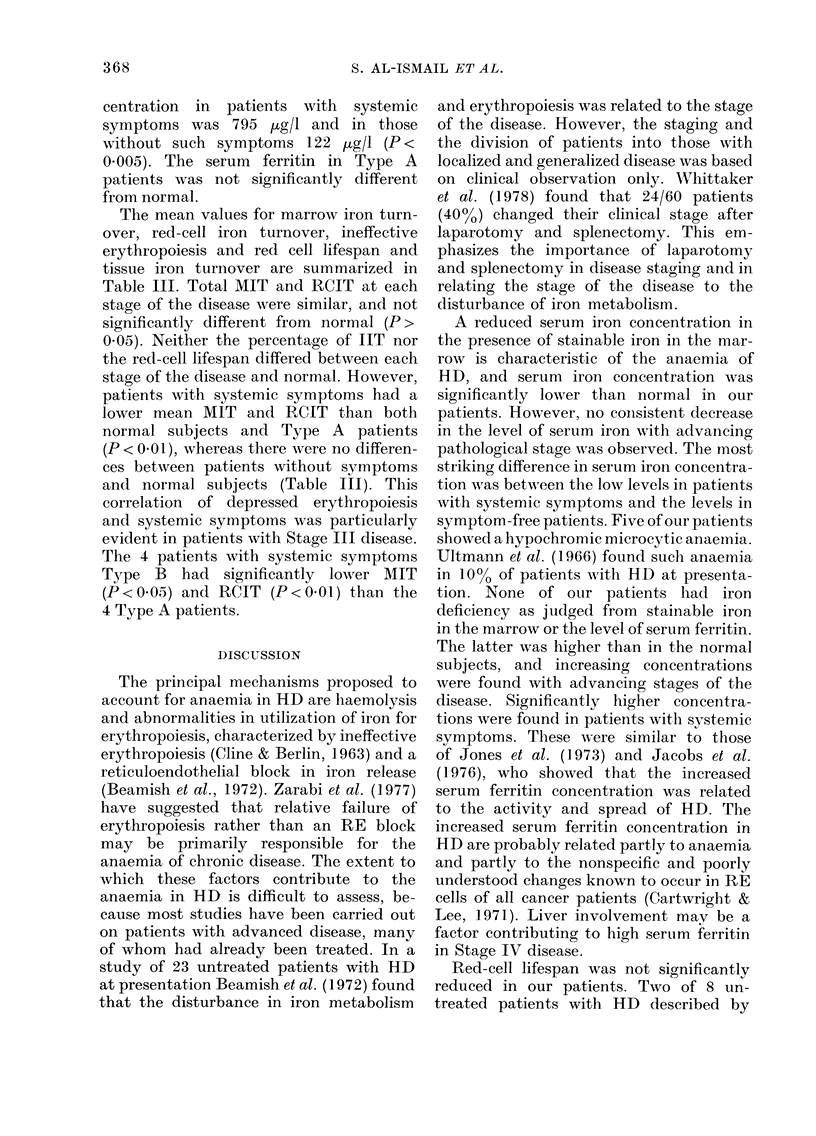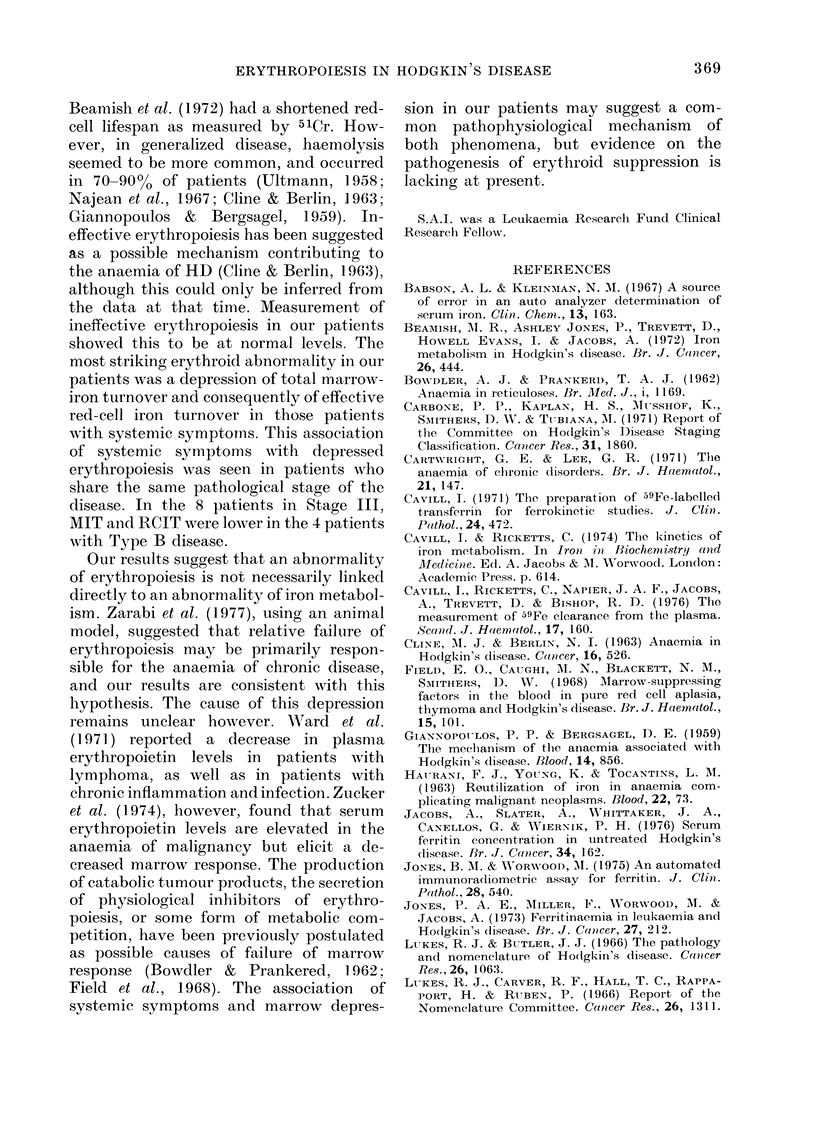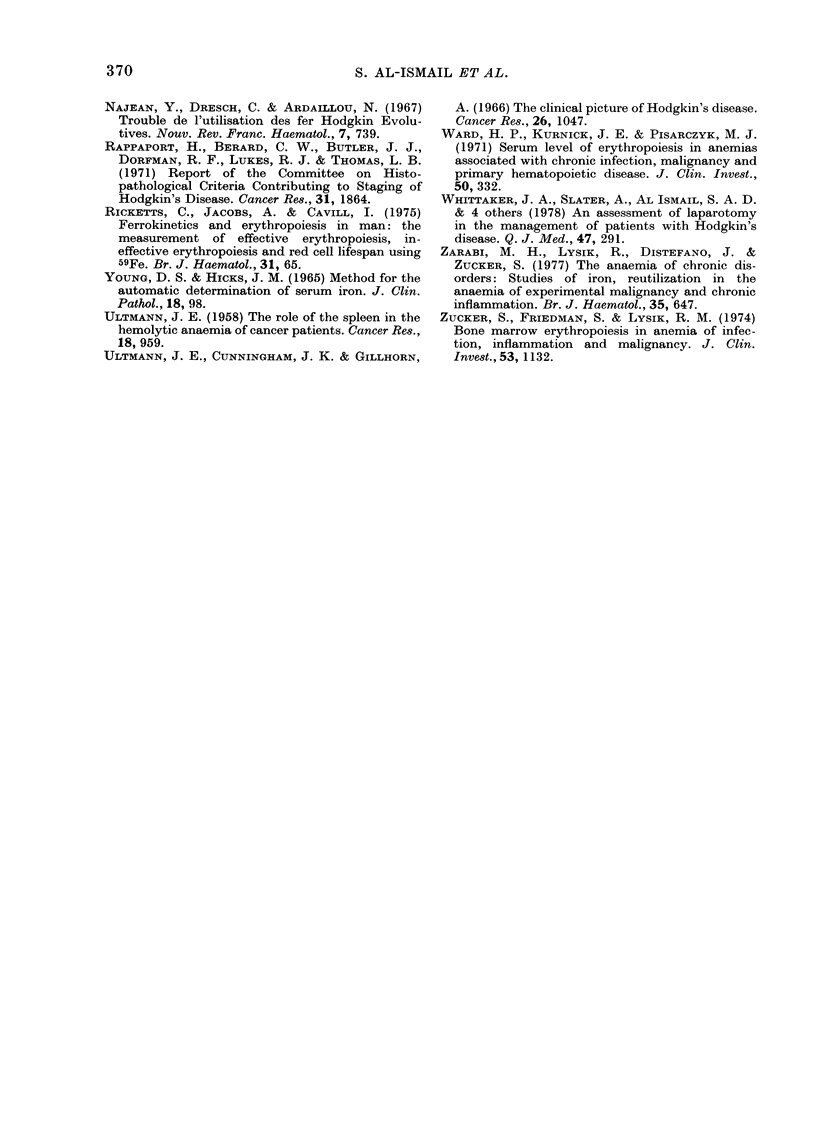# Erythropoiesis and iron metabolism in Hodgkin's disease.

**DOI:** 10.1038/bjc.1979.190

**Published:** 1979-09

**Authors:** S. Al-Ismail, I. Cavill, I. H. Evans, A. Jacobs, C. Ricketts, D. Trevett, J. A. Whittaker

## Abstract

Recently developed techniques for the investigation of iron kinetics were used to study the disturbance of iron metabolism in 19 untreated patients with Hodgkin's diseases (HD). The erythroid abnormality in newly diagnosed HD appears to be confined to those patients with systemic symptoms of weight loss, night sweats and fever, and consists of depression of marrow erythroid activity. These patients had a significnatly lower haemoglobin and serum iron concentration and a higher serum ferritin concentration, both when compared to normal subjects and to those patients with HD who lacked systemic symptoms. Ineffective erythropoiesis and red-cell destruction were not significantly increased. The present findings, confirm that HD patients with systemic symptoms have a depression of erythropoiesis, and that in these patients the marrow fails to respond to the stimulus of mild anaemia.


*Br. J. Cancer* (1979) **40,** 365

### ERYTHROPOIESIS AND IRON METABOLISM IN HODGKIN'S DISEASE

S. AL-ISMAIL*, I. CAVILL*, I. H. EVANS†, A. JACOBS*, C. RICKETTS*, D. TREVETT* AND J. A. WHITTAKER

*From the *Department of Haematology, Welsh National School of Medicine, University Hospital of Wales, and the † Velindre Hospital, Cardiff*

Received 21 March 1979 Accepted 14 May 1979

**Summary.—Recently developed techniques for the investigation of iron kinetics were used to study the disturbance of iron metabolism in 19 untreated patients with Hodgkin's diseases (HD). The erythroid abnormality in newly diagnosed HD appears to be confined to those patients with systemic symptoms of weight loss, night sweats and fever, and consists of depression of marrow erythroid activity. These patients had a significantly lower haemoglobin and serum iron concentration and a higher serum ferritin concentration, both when compared to normal subjects and to those patients with HD who lacked systemic symptoms. Ineffective erythropoiesis and red-cell destruction were not significantly increased. The present findings confirm that HD patients with systemic symptoms have a depression of erythropoiesis, and that in these patients the marrow fails to respond to the stimulus of mild anaemia.**

THE MECHANISM of anaemia in Hodgkin's disease (HD) is unclear. Abnormalities of iron utilization have been noted on many occasions in the past (Giannopoulos & Bergsagel 1959; Haurani *et al.*, 1963) and ineffective erythropoiesis (Cline & Berlin 1963). Beamish *et al.* (1972) suggested that impairment of iron release from the reticuloendothelial (RE) system with a consequent failure of iron delivery to the marrow was common in HD. Many of the patients investigated in these earlier studies had already undergone treatment with radiation and chemotherapy. In addition, the ferrokinetic methods used did not provide a quantitative assessment of effective and ineffective red-cell production (Cavill & Ricketts, 1974).

The purpose of the preesnt study was to re-evaluate and define the abnormalities of iron metabolism and the possible mechanism of anaemia in patients presenting with HD. Ferrokinetic methods allowing quantitative estimates of erythropoiesis were used (Ricketts *et al.*, 1975).

### PATIENTS

Nineteen newly diagnosed untreated patients with HD and 14 healthy adults were studied. Fully informed consent was obtained in all cases. There were 14 males and 5 female patients and their ages ranged from 17 to 69 years with a mean of 43 years. None of the patients was receiving any form of medical treatment at the time of investigation. The diagnosis of HD was based on lymphnode biopsy in all patients, except one in whom the diagnosis was made after splenectomy. Routine haematological and biochemical investigations, marrow aspiration and trephine biopsy, chest X-ray and hilar tomography, radiological skeletal survey and bipedal lymphangiography were carried out in all patients.

The stage of the disease was assessed before laparotomy according to the Ann Arbor International Convention (Carbone *et al.*, 1971). All patients except those with biopsy-proven Stage IV disease then underwent laparotomy and splenectomy with open liver biopsy. The histological classification was according to Lukes & Butler (1966) as modified at Rye (Lukes *et al.*, 1966) and Ann Arbor (Rapport *et al.*, 1971) International Conventions. The patients were classified as Type A or B on the basis of the absence or presence of systemic symptoms of unexplained fever (more than 38°C), weight loss of more than 10% during the 6 months preceding the diagnosis, or night sweats (Carbone *et al.*, 1971).

### METHODS

Haemoglobin concentration and mean corpuscular volume (MCV) were measured with a Coulter Counter Model S. Serum iron concentration and total iron-binding capacity were measured according to the method of Young & Hicks (1965) as modified by Babson & Kleinmen (1967). The method of Jones & Worwood (1975) was used to measure the serum ferritin concentration.

Radioisotope studies were carried out at presentation, before laparotomy. A sample of each subject's plasma transferrin was specifically labelled with $^{59}Fe$ (Cavill, 1971) and the plasma $^{59}Fe$ clearance curve was defined over 10–14 days by the method of Cavill *et al.* (1976). The results were analysed as described by Ricketts *et al.* (1975) and the following parameters were calculated: total erythroid marrow iron turnover (MIT), effective red-cell iron turnover (RCIT), and ineffective iron turnover (IIT). The mean cell lifespan and mean red-cell production were calculated from red-cell iron turnover. Ineffective iron turnover was expressed as a percentage of MIT.

The differences between means were assessed using a one-way analysis of variance, whilst correlation between parameters was assessed using the Pearson product-moment correlation coefficient.

### RESULTS

The number of patients showing different stages of the disease, histological classes and the presence or absence of symptoms is shown in Table I. The haematological and iron status of the subjects are summarized in Table II. Only 5 of the patients were anaemic (haemoglobin concentration < 13 g/dl for men or < 12 g/dl for women) but 9 patients showed a microcytic blood picture (MCV < 84 fl). There was no significant difference in the haemoglobin concentration or MCV between the 4 stages of the disease, nor did they differ from the control group. However, patients with systemic symptoms had lower haemoglobin concentrations and lower MCVs compared both to the normal subjects and to the patients without such symptoms ($P < 0{\cdot}005$).

TABLE I.—*The distribution of* 19 *untreated patients with Hodgkin's disease* (*HD*) *in relation to symptoms, stage of disease and histological classification*

| Histological type | Stage of disease I | II | III | IV |
|---|---|---|---|---|
| Lymphocytic predominant | 2 | 1 | – | – |
| Nodular sclerosing | 2 | 1 | 4 | – |
| Mixed cellularity | 2 | – | 4 | 1 |
| Lymphocyte depleted | – | 1 | – | 1 |
| Type A | 6 | 2 | 4 | – |
| Type B | – | 1 | 4 | 2 |

There was a significant correlation between MCV and serum iron concentration ($r = 0{\cdot}58$, $P < 0{\cdot}01$) and sideropenia was predominantly associated with the presence of systemic symptoms (Table III). Patients with systemic symptoms had a mean serum iron concentration which was significantly lower ($P < 0{\cdot}005$) than both that of the patients without such symptoms and that of the normal group. Although serum iron varied between the 4 stages of the disease, there was no clear relationship with advancing stage. Serum ferritin was significantly higher in the patients with HD than in the control group, and significantly higher concentrations ($P < 0{\cdot}01$) were found with advancing stages of the disease (Table III). There was a significant negative correlation between the serum iron concentration and serum ferritin concentration ($r = -0{\cdot}57$, $P < 0{\cdot}02$); patients with low serum iron concentrations had high ferritin concentrations. This appears to be related both to the presence of anaemia and systemic symptoms. The mean serum ferritin concentration in the non-anaemic patients was 185 $\mu$g/l and the mean value in the anaemic patients was 776 $\mu$g/l ($P < 0{\cdot}01$); the mean serum ferritin con-

TABLE II.—*Haematological amd iron status (mean and range) of* 14 *normal subjects and* 19 *patients with HD*

| | | HD | | | | | | |
|---|---|---|---|---|---|---|---|---|
| | | Stage of disease | | | | | Type | |
| | Normal | I | II | III | IV | Total | A | B |
| Haemoglobin concentration g/dl | 14·5 (11·9–17·1) | 14·7 (11·9–16·3) | 13·1 (12·2–14·7) | 13·2 (9·9–15·5) | 11·6 (10·7–12·5) | 13·5 (9·9–16·3) | 14·4 (11·9–16·3) | 11·9 (9·9–13·3) |
| Mean corpuscular volume fl | 87·7 (84–92) | 88·6 (80–98) | 86·3 (80–92) | 84·1 (75–96) | 79·5 (78–81) | 85 (75–98) | 88 (80–98) | 81 (75–90) |
| Serum iron μmol/l | 19·5 (10–34) | 19·9 (12·5–26·5) | 5·4 (4·5–6·6) | 12·3 (2·3–29·5) | 4·5 (4–5) | 12·7 (2·3–29·5) | 17·8 (5–29·5) | 4 (2·3–6) |
| Serum ferritin μg/l | 104 (14–379) | 70 (19–214) | 194·7 (108–265) | 416·6 (64–920) | 1319·5 (1070–1569) | 370 (19–1569) | 122 (19–280) | 795 (265–1569) |

TABLE III.—*Erythropoietic activity and red-cell lifespan (mean and range) in* 14 *normal subjects and* 19 *patients with HD*

| | | HD | | | | | | |
|---|---|---|---|---|---|---|---|---|
| | | Stage of disease | | | | | Type | |
| | Normal | I | II | III | IV | Total | A | B |
| Marrow iron turnover (MOT) μmol/l blood/day | 110 (73–142) | 129 (108–158) | 86·7 (68–114) | 102 (56–178) | 94 (79–109) | 106 (56–178) | 123·6 (68–178) | 80·7 (56–109) |
| Red cell iron turnover (RCIT) μmol/l blood/day | 85 (54–124) | 102 (78–120) | 65·7 (53–79) | 78·4 (36–115) | 79 (68–90) | 83·8 (36–120) | 96 (53–120) | 62 (36–90) |
| Ineffective iron turnover (IIT) % of MIT | 22 (13–33) | 20·7 (14–33) | 23 (17–31) | 23 (6–36) | 15·5 (14–17) | 22 (6–36) | 21 (6–35) | 23 (14–36) |
| Red-cell life span, days | 99 (70–153) | 83 (72–96) | 112·3 (83–154) | 92 (60–143) | 68 (73–85) | 91 (60–154) | 86·8 (60–154) | 99 (73–143) |

centration in patients with systemic symptoms was 795 $\mu g/l$ and in those without such symptoms 122 $\mu g/l$ ($P < 0{\cdot}005$). The serum ferritin in Type A patients was not significantly different from normal.

The mean values for marrow iron turnover, red-cell iron turnover, ineffective erythropoiesis and red cell lifespan and tissue iron turnover are summarized in Table III. Total MIT and RCIT at each stage of the disease were similar, and not significantly different from normal ($P > 0{\cdot}05$). Neither the percentage of IIT nor the red-cell lifespan differed between each stage of the disease and normal. However, patients with systemic symptoms had a lower mean MIT and RCIT than both normal subjects and Type A patients ($P < 0{\cdot}01$), whereas there were no differences between patients without symptoms and normal subjects (Table III). This correlation of depressed erythropoiesis and systemic symptoms was particularly evident in patients with Stage III disease. The 4 patients with systemic symptoms Type B had significantly lower MIT ($P < 0{\cdot}05$) and RCIT ($P < 0{\cdot}01$) than the 4 Type A patients.

### DISCUSSION

The principal mechanisms proposed to account for anaemia in HD are haemolysis and abnormalities in utilization of iron for erythropoiesis, characterized by ineffective erythropoiesis (Cline & Berlin, 1963) and a reticuloendothelial block in iron release (Beamish *et al.*, 1972). Zarabi *et al.* (1977) have suggested that relative failure of erythropoiesis rather than an RE block may be primarily responsible for the anaemia of chronic disease. The extent to which these factors contribute to the anaemia in HD is difficult to assess, because most studies have been carried out on patients with advanced disease, many of whom had already been treated. In a study of 23 untreated patients with HD at presentation Beamish *et al.* (1972) found that the disturbance in iron metabolism and erythropoiesis was related to the stage of the disease. However, the staging and the division of patients into those with localized and generalized disease was based on clinical observation only. Whittaker *et al.* (1978) found that 24/60 patients (40%) changed their clinical stage after laparotomy and splenectomy. This emphasizes the importance of laparotomy and splenectomy in disease staging and in relating the stage of the disease to the disturbance of iron metabolism.

A reduced serum iron concentration in the presence of stainable iron in the marrow is characteristic of the anaemia of HD, and serum iron concentration was significantly lower than normal in our patients. However, no consistent decrease in the level of serum iron with advancing pathological stage was observed. The most striking difference in serum iron concentration was between the low levels in patients with systemic symptoms and the levels in symptom-free patients. Five of our patients showed a hypochromic microcytic anaemia. Ultmann *et al.* (1966) found such anaemia in 10% of patients with HD at presentation. None of our patients had iron deficiency as judged from stainable iron in the marrow or the level of serum ferritin. The latter was higher than in the normal subjects, and increasing concentrations were found with advancing stages of the disease. Significantly higher concentrations were found in patients with systemic symptoms. These were similar to those of Jones *et al.* (1973) and Jacobs *et al.* (1976), who showed that the increased serum ferritin concentration was related to the activity and spread of HD. The increased serum ferritin concentration in HD are probably related partly to anaemia and partly to the nonspecific and poorly understood changes known to occur in RE cells of all cancer patients (Cartwright & Lee, 1971). Liver involvement may be a factor contributing to high serum ferritin in Stage IV disease.

Red-cell lifespan was not significantly reduced in our patients. Two of 8 untreated patients with HD described by

Beamish *et al.* (1972) had a shortened red-cell lifespan as measured by $^{51}$Cr. However, in generalized disease, haemolysis seemed to be more common, and occurred in 70–90% of patients (Ultmann, 1958; Najean *et al.*, 1967; Cline & Berlin, 1963; Giannopoulos & Bergsagel, 1959). Ineffective erythropoiesis has been suggested as a possible mechanism contributing to the anaemia of HD (Cline & Berlin, 1963), although this could only be inferred from the data at that time. Measurement of ineffective erythropoiesis in our patients showed this to be at normal levels. The most striking erythroid abnormality in our patients was a depression of total marrow-iron turnover and consequently of effective red-cell iron turnover in those patients with systemic symptoms. This association of systemic symptoms with depressed erythropoiesis was seen in patients who share the same pathological stage of the disease. In the 8 patients in Stage III, MIT and RCIT were lower in the 4 patients with Type B disease.

Our results suggest that an abnormality of erythropoiesis is not necessarily linked directly to an abnormality of iron metabolism. Zarabi *et al.* (1977), using an animal model, suggested that relative failure of erythropoiesis may be primarily responsible for the anaemia of chronic disease, and our results are consistent with this hypothesis. The cause of this depression remains unclear however. Ward *et al.* (1971) reported a decrease in plasma erythropoietin levels in patients with lymphoma, as well as in patients with chronic inflammation and infection. Zucker *et al.* (1974), however, found that serum erythropoietin levels are elevated in the anaemia of malignancy but elicit a decreased marrow response. The production of catabolic tumour products, the secretion of physiological inhibitors of erythropoiesis, or some form of metabolic competition, have been previously postulated as possible causes of failure of marrow response (Bowdler & Prankered, 1962; Field *et al.*, 1968). The association of systemic symptoms and marrow depression in our patients may suggest a common pathophysiological mechanism of both phenomena, but evidence on the pathogenesis of erythroid suppression is lacking at present.

S.A.I. was a Leukaemia Research Fund Clinical Research Fellow.

### REFERENCES

BABSON, A. L. & KLEINMAN, N. M. (1967) A source of error in an auto analyzer determination of serum iron. *Clin. Chem.*, **13**, 163.

BEAMISH, M. R., ASHLEY JONES, P., TREVETT, D., HOWELL EVANS, I. & JACOBS, A. (1972) Iron metabolism in Hodgkin's disease. *Br. J. Cancer*, **26**, 444.

BOWDLER, A. J. & PRANKERD, T. A. J. (1962) Anaemia in reticuloses. *Br. Med. J.*, i, 1169.

CARBONE, P. P., KAPLAN, H. S., MUSSHOF, K., SMITHERS, D. W. & TUBIANA, M. (1971) Report of the Committee on Hodgkin's Disease Staging Classification. *Cancer Res.*, **31**, 1860.

CARTWRIGHT, G. E. & LEE, G. R. (1971) The anaemia of chronic disorders. *Br. J. Haematol.*, **21**, 147.

CAVILL, I. (1971) The preparation of $^{59}$Fe-labelled transferrin for ferrokinetic studies. *J. Clin. Pathol.*, **24**, 472.

CAVILL, I. & RICKETTS, C. (1974) The kinetics of iron metabolism. In *Iron in Biochemistry and Medicine*. Ed. A. Jacobs & M. Worwood. London: Academic Press. p. 614.

CAVILL, I., RICKETTS, C., NAPIER, J. A. F., JACOBS, A., TREVETT, D. & BISHOP, R. D. (1976) The measurement of $^{59}$Fe clearance from the plasma. *Scand. J. Haematol.*, **17**, 160.

CLINE, M. J. & BERLIN, N. I. (1963) Anaemia in Hodgkin's disease. *Cancer*, **16**, 526.

FIELD, E. O., CAUGHI, M. N., BLACKETT, N. M., SMITHERS, D. W. (1968) Marrow-suppressing factors in the blood in pure red cell aplasia, thymoma and Hodgkin's disease. *Br. J. Haematol.*, **15**, 101.

GIANNOPOULOS, P. P. & BERGSAGEL, D. E. (1959) The mechanism of the anaemia associated with Hodgkin's disease. *Blood*, **14**, 856.

HAURANI, F. J., YOUNG, K. & TOCANTINS, L. M. (1963) Reutilization of iron in anaemia complicating malignant neoplasms. *Blood*, **22**, 73.

JACOBS, A., SLATER, A., WHITTAKER, J. A., CANELLOS, G. & WIERNIK, P. H. (1976) Serum ferritin concentration in untreated Hodgkin's disease. *Br. J. Cancer*, **34**, 162.

JONES, B. M. & WORWOOD, M. (1975) An automated immunoradiometric assay for ferritin. *J. Clin. Pathol.*, **28**, 540.

JONES, P. A. E., MILLER, F., WORWOOD, M. & JACOBS, A. (1973) Ferritinaemia in leukaemia and Hodgkin's disease. *Br. J. Cancer*, **27**, 212.

LUKES, R. J. & BUTLER, J. J. (1966) The pathology and nomenclature of Hodgkin's disease. *Cancer Res.*, **26**, 1063.

LUKES, R. J., CARVER, R. F., HALL, T. C., RAPPAPORT, H. & RUBEN, P. (1966) Report of the Nomenclature Committee. *Cancer Res.*, **26**, 1311.

NAJEAN, Y., DRESCH, C. & ARDAILLOU, N. (1967) Trouble de l'utilisation des fer Hodgkin Evolutives. *Nouv. Rev. Franc. Haematol.*, **7**, 739.

RAPPAPORT, H., BERARD, C. W., BUTLER, J. J., DORFMAN, R. F., LUKES, R. J. & THOMAS, L. B. (1971) Report of the Committee on Histopathological Criteria Contributing to Staging of Hodgkin's Disease. *Cancer Res.*, **31**, 1864.

RICKETTS, C., JACOBS, A. & CAVILL, I. (1975) Ferrokinetics and erythropoiesis in man: the measurement of effective erythropoiesis, ineffective erythropoiesis and red cell lifespan using $^{59}Fe$. *Br. J. Haematol.*, **31**, 65.

YOUNG, D. S. & HICKS, J. M. (1965) Method for the automatic determination of serum iron. *J. Clin. Pathol.*, **18**, 98.

ULTMANN, J. E. (1958) The role of the spleen in the hemolytic anaemia of cancer patients. *Cancer Res.*, **18**, 959.

ULTMANN, J. E., CUNNINGHAM, J. K. & GILLHORN, A. (1966) The clinical picture of Hodgkin's disease. *Cancer Res.*, **26**, 1047.

WARD, H. P., KURNICK, J. E. & PISARCZYK, M. J. (1971) Serum level of erythropoiesis in anemias associated with chronic infection, malignancy and primary hematopoietic disease. *J. Clin. Invest.*, **50**, 332.

WHITTAKER, J. A., SLATER, A., AL ISMAIL, S. A. D. & 4 others (1978) An assessment of laparotomy in the management of patients with Hodgkin's disease. *Q. J. Med.*, **47**, 291.

ZARABI, M. H., LYSIK, R., DISTEFANO, J. & ZUCKER, S. (1977) The anaemia of chronic disorders: Studies of iron, reutilization in the anaemia of experimental malignancy and chronic inflammation. *Br. J. Haematol.*, **35**, 647.

ZUCKER, S., FRIEDMAN, S. & LYSIK, R. M. (1974) Bone marrow erythropoiesis in anemia of infection, inflammation and malignancy. *J. Clin. Invest.*, **53**, 1132.